# Impact of Gel-Derived Morphology-Controlled UiO-66/Cellulose Nanofiber Composite Separators on the Performance of Aqueous Zinc-Ion Batteries

**DOI:** 10.3390/gels12010075

**Published:** 2026-01-15

**Authors:** Tian Zhao, Jiangrong Yu, Shilin Peng, Yan Wu, Tianhang Wang, Zhuoheng Li, Ling Shen, Christoph Janiak, Yi Chen

**Affiliations:** 1 School of Materials Science and Engineering, Hunan University of Technology, Zhuzhou 412007, China; 18593328326@163.com (J.Y.); psl18670833816@163.com (S.P.); wuyan529713@163.com (Y.W.); wth17803229131@163.com (T.W.); 13873515101@163.com (Z.L.); 2 Center for Materials Research and Analysis, Wuhan University of Technology, Wuhan 430070, China; lingshen@whut.edu.cn; 3 Institut für Anorganische Chemie und Strukturchemie, Heinrich-Heine-Universität Düsseldorf, Universitätsstr. 1, D-40225 Düsseldorf, Germany; janiak@uni-duesseldorf.de

**Keywords:** aqueous zinc-ion batteries, UiO-66, cellulose nanofiber gel, battery separator, suppressing zinc dendrites

## Abstract

Zinc dendrite growth and side reactions remain critical challenges hindering the advancement of aqueous zinc-ion batteries (AZIBs). This study proposes a gel-based strategy for designing high-performance separators by regulating the crystal morphology of the metal–organic framework UiO-66 within a cellulose nanofiber (CNF) gel matrix. The resulting gel-derived separators exhibit distinctive structural and interfacial properties that significantly enhance battery performance. Compared with hierarchical porous structures (H-UiO-66), the octahedral morphology (O-UiO-66) disperses more uniformly in the CNF gel network, forming well-defined ion transport channels through its integrated gel architecture. The fabricated O-UiO-66/CNF gel separator demonstrates exceptional hydrophilicity (contact angle 21°), high porosity (73.2%), and significantly improved zinc ion migration number (0.72). Electrochemical tests reveal that this gel-based separator effectively guides uniform zinc deposition while suppressing dendrite growth. Zn/Zn symmetric cells using the O-UiO-66/CNF gel separator achieve a cycle life exceeding 800 h at 1 mA cm^−2^. The Zn/MnO_2_ full cell maintains 98.1% capacity retention after 100 cycles at 1 A g^−1^. This work establishes a structure–performance relationship between MOF morphology and gel separator properties, providing new insights for designing advanced gel-based materials for AZIBs.

## 1. Introduction

Lithium-ion batteries (LIBs), the cornerstone of modern electrochemical energy storage, dominate portable electronics and electric vehicle markets due to their high energy density and long cycle life [1,2,3]. However, their application in large-scale grid storage is constrained by the limited global distribution of lithium resources [4] and safety concerns stemming from flammable organic electrolytes [5,6]. Furthermore, the energy density of current mainstream cathode materials is approaching its theoretical limit (approximately 350 Wh/kg for NMC systems) [7]. While solid-state batteries offer a promising path to higher safety and energy density, their widespread commercialization is still impeded by interfacial instability and high manufacturing costs [8,9]. In this context, aqueous zinc-ion batteries (AZIBs) have emerged as a compelling alternative for stationary storage [10], leveraging zinc’s natural abundance [11], inherent safety of aqueous electrolytes, and high volumetric capacity [12].

The development of high-performance separators is critical to overcoming the challenges of zinc dendrite growth and parasitic reactions in AZIBs [13,14,15]. Cellulose nanofibers (CNFs) gel matrix provide a robust, hydrophilic scaffold [16], while metal–organic frameworks (MOFs) offer precise ion-sieving capabilities via their tunable pore structures [17,18,19]. UiO-66 [20], a zirconium-based MOF, is particularly attractive due to its exceptional chemical stability and well-defined microporous channels [21,22]. Previous studies, including work by Zhao et al. [23], demonstrate that the carboxylate groups on UiO-66 can effectively coordinate with water molecules, reducing free water activity and mitigating side reactions [24,25]. Furthermore, when the pore size is commensurate with the hydrated Zn^2+^ ion (~0.8 nm), UiO-66 can homogenize ion flux and guide uniform deposition [26,27].

This study addresses key challenges in the design of high-performance separators for aqueous zinc-ion batteries (AZIBs). Although UiO-66/cellulose composites show promise, the field lacks a fundamental understanding of how the morphology of the integrated metal–organic framework (MOF) dictates the final separator’s properties. Specifically, it remains unclear how different UiO-66 architectures influence the gel network formation within a cellulose nanofiber (CNF) matrix, and what mechanistic role this plays in ion transport and zinc deposition uniformity. To bridge these knowledge gaps, we developed a novel gel-based separator through the in situ incorporation of morphology-engineered UiO-66 into a CNF matrix. Our findings reveal that an octahedral UiO-66 morphology (O-UiO-66) facilitates the formation of a more homogeneous and interconnected gel network compared to a hierarchical porous structure (H-UiO-66). This tailored network results in superior ion transport channels, enhanced electrolyte wettability, and improved interfacial stability. Through comprehensive analysis, we establish clear correlations between the MOF morphology, the nanoscale gel structure, and the macroscopic electrochemical performance. This work underscores the critical importance of precise morphological control in gel-MOF composites and provides a rational design principle for advanced functional separators, offering new insights into the development of durable and efficient energy storage systems.

## 2. Results and Discussion

**Figure 1:**
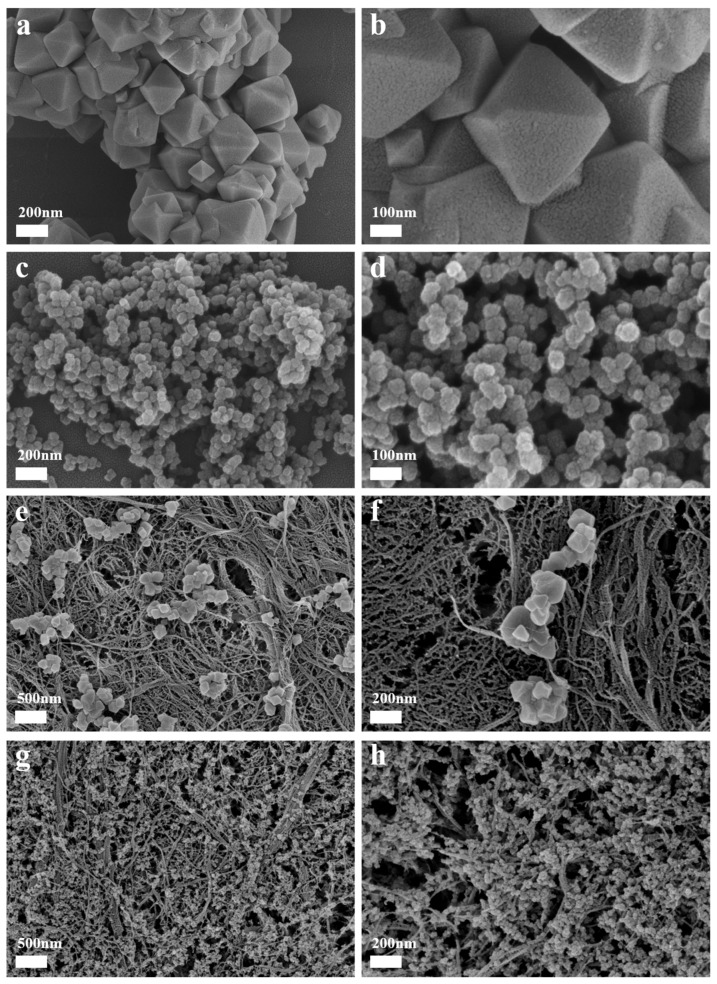
SEM images of UiO-66 with different morphological structures: (**a**,**b**) O-UiO-66 showing regular octahedral morphology with particle size of ~300 nm and good dispersion; (**c**,**d**) H-UiO-66 exhibiting irregular granular morphology with particle size of ~50 nm and visible aggregation; (**e**,**f**) O-UiO-66@CNF separator showing uniform distribution of octahedral particles on and within CNF fibers, forming well-defined ion transport channels; (**g**,**h**) H-UiO-66@CNF separator displaying significant particle aggregation on fiber surfaces, which may lead to uneven pore structure and ion transport efficiency. The octahedral morphology facilitates better integration with the cellulose nanofiber network compared to the hierarchical porous structure.

In this work, two types of UiO-66 with distinct morphologies were synthesized via a two-step solvothermal method, and their SEM images are shown in Figure 1a–d. Figure 1a,b display the micro-morphology of the O-UiO-66 sample, revealing a regular and uniform octahedral structure with a particle size of approximately 300 nm and relatively good dispersion. Figure 1c,d present the micro-morphology of the H-UiO-66 sample, which exhibits an irregular granular morphology with a particle size of about 50 nm. Some crystal grains show aggregation and stacking phenomena. This is attributed to the increased solution volume, which weakens Brownian motion and reduces effective molecular collisions, leading to morphological defects and a decrease in crystal size [28,29].

Figure 1e–h show surface SEM images of the composite separators incorporating UiO-66 with different morphologies. The images clearly reveal numerous UiO-66 particles on the fiber surfaces, confirming the successful loading of UiO-66 onto the CNF fibers. This observation further verifies the effective combination of UiO-66 and CNF, laying a foundation for subsequent electrochemical performance optimization. Figure 1g,h depict the micro-morphology of the H-UiO-66@CNF separator. The images indicate significant particle aggregation, with these particles primarily adhering to the surface of the fiber strands. This agglomeration may lead to uneven particle distribution, consequently affecting the separator’s pore structure and ion transport efficiency.

In contrast, the images of the O-UiO-66@CNF separator in Figure 1e,f demonstrate better dispersion of the octahedral particles. The particles are not only uniformly distributed on the fiber surfaces but also penetrate into the fiber network. This homogeneous dispersion helps create a more consistent pore environment, thereby promoting uniform zinc ion transport and deposition processes within the separator [22,23].

Statistical analysis of particle size distributions (Figure S2) based on SEM measurements of 200 particles confirms that O-UiO-66 exhibits a relatively narrow size distribution centered at 298 ± 45 nm, while H-UiO-66 shows a broader distribution with a mean particle size of 52 ± 18 nm. The uniform particle size of O-UiO-66 contributes to its better dispersion within the CNF network, while the polydisperse nature of H-UiO-66, combined with its smaller primary particle size, promotes aggregation due to higher surface energy.

The SEM images reveal distinct differences in the distribution patterns of the two MOF morphologies within the CNF network. For H-UiO-66@CNF (Figure 1g,h), the smaller particle size (50 nm) results in higher particle number density on the CNF fiber surfaces, creating the appearance of dense and homogeneous decoration when viewed in two-dimensional SEM projections. This high surface density might be interpreted as improved dispersion at first glance. However, closer examination reveals that the H-UiO-66 particles exist primarily as surface-adsorbed aggregates rather than being uniformly distributed throughout the three-dimensional CNF network. The high surface energy of the smaller nanoparticles drives them to aggregate and adsorb onto the readily available fiber surfaces during the mixing and filtration process.

In contrast, the O-UiO-66@CNF separator (Figure 1e,f) exhibits a different distribution pattern where the larger octahedral particles (300 nm) are not only present on fiber surfaces but are also effectively embedded within the interstitial spaces between fibers throughout the separator thickness. This three-dimensional distribution is achieved because the larger, well-defined octahedral particles have lower surface energy and better colloidal stability, allowing them to distribute more uniformly throughout the CNF gel matrix before filtration. The octahedral morphology also facilitates better mechanical interlocking with the flexible CNFs, preventing particle migration during processing. Consequently, while the H-UiO-66 shows higher particle density on surface fibers, the O-UiO-66 achieves more uniform three-dimensional dispersion throughout the separator volume, which is more beneficial for creating consistent ion transport pathways and uniform current distribution across the entire electrode area. This distinction between surface decoration and three-dimensional dispersion is critical for understanding the superior performance of the O-UiO-66@CNF separator.

In zinc batteries, efficient ion transport is crucial for the charge–discharge processes [30]. This structural difference significantly impacts battery performance [31]. The agglomeration of particles in the H-UiO-66@CNF separator may lead to localized ion transport blockage, adversely affecting the battery’s charge–discharge efficiency and cycling stability. Conversely, the uniform distribution of particles in the O-UiO-66@CNF separator facilitates the formation of continuous ion transport channels, thereby enhancing the overall battery performance. Theoretically, well-dispersed MOF particles are more conducive to uniform Zn^2+^ transport and long-term, stable deposition processes [12,32,33]. Uniform dispersion prevents the formation of particle-free regions with high local current density (hot spots) and particle-rich regions that can block ion transport, thereby ensuring consistent ion flux across the entire separator area.

**Figure 2:**
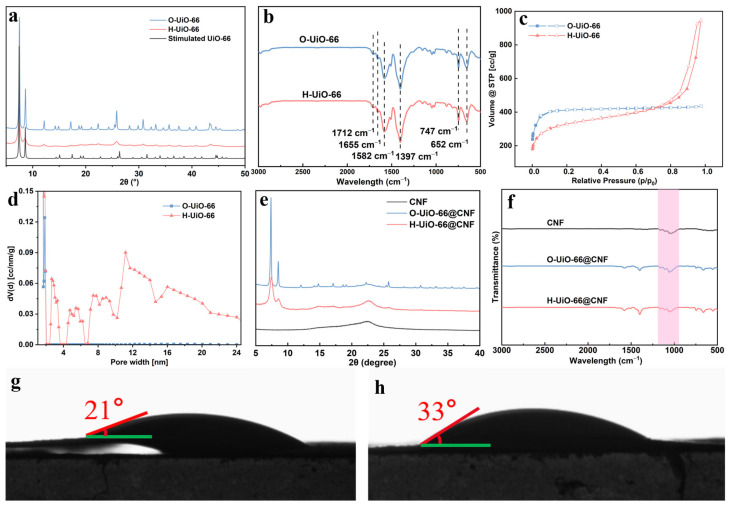
(**a**) XRD patterns of O-UiO-66 and H-UiO-66 powders, both matching the simulated UiO-66 standard pattern, with O-UiO-66 showing higher crystallinity; (**b**) FTIR spectra confirming successful UiO-66 synthesis through characteristic Zr-O peaks at 652 and 747 cm^−1^ and C=O stretching vibrations; (**c**) N_2_ adsorption–desorption isotherms showing type I behavior for both samples, with H-UiO-66 exhibiting additional type IV hysteresis loop indicating mesoporous structure; (**d**) Pore size distribution revealing microporous characteristics of both samples with H-UiO-66 showing additional mesopores; (**e**) XRD patterns of composite separators preserving characteristic peaks of both UiO-66 and CNF (2θ = 22.5°), confirming successful embedding without impurity formation; (**f**) FTIR spectra of separators retaining characteristic peaks of both CNF and UiO-66 without new chemical bond signals, indicating effective physical combination; (**g**) Contact angle of 21° for O-UiO-66@CNF separator; (**h**) Contact angle of 33° for H-UiO-66@CNF separator, both demonstrating superior hydrophilicity compared to pure CNF separator (48°).

As shown in Figure 2a, both O-UiO-66 and H-UiO-66 exhibit diffraction peaks at 2θ values of 7.3°, 8.5°, 12.1°, 18.6°, and 25.6°, which match the simulated UiO-66 standard pattern (reference to Crystallography Open Database entry COD-9008985, corresponding to the original UiO-66 structure reported by Cavka et al. [20]). The peak at 7.3° corresponds to the (111) plane, 8.5° to the (002) plane, 12.1° to the (022) plane, 18.6° to the (004) plane, and 25.6° to the (224) plane of the cubic UiO-66 structure (space group Fm-3m). These assignments confirm that both samples are pure-phase UiO-66 with the expected fcu topology. Furthermore, the height and sharpness of the diffraction peaks indicate that O-UiO-66 presents sharp and high-intensity characteristic peaks, suggesting a highly ordered crystalline structure. In contrast, the diffraction peaks of H-UiO-66 are significantly broadened, indicating lower crystallinity. This result is consistent with the SEM observations.

Figure 2b shows the FTIR spectra of the UiO-66 samples. The characteristic Zr-O peaks are observed at 652 cm^−1^ and 747 cm^−1^. The peaks centered at 1397 cm^−1^, 1582 cm^−1^, 1655 cm^−1^, and 1712 cm^−1^ correspond to the symmetric stretching vibrations of C=O in the aromatic benzene rings. The peak at 1506 cm^−1^ is attributed to the C=C vibration within the benzene rings. These findings further confirm the successful synthesis of UiO-66 with both morphologies.

Figure 2c,d present the N_2_ adsorption–desorption isotherms and pore size distribution curves of the two samples, respectively, with the basic pore structure parameters summarized in Table 1. The pore structure parameters summarized in Table 1 reveal important differences between the two morphologies. While O-UiO-66 possesses a larger specific surface area (1589.4 m^2^·g^−1^) and higher micropore area (1847.8 m^2^·g^−1^), H-UiO-66 exhibits a larger total pore volume (1.46 cm^3^·g^−1^) due to the presence of both micropores and mesopores. The pore structure of O-UiO-66 (with a total pore volume of 0.67 cm^3^·g^−1^) is dominated by micropores, which may be well-matched to specific ion sizes (e.g., hydrated ions) to enable efficient ion sieving. In contrast, the larger total pore volume of H-UiO-66 (1.46 cm^3^·g^−1^) reflects its pore structure characteristics, which may compromise its molecular sieving capability while enhancing bulk electrolyte uptake. Both samples exhibit type I adsorption isotherms according to the IUPAC classification, indicating their microporous characteristics [34]. Notably, the isotherm of H-UiO-66 shows a distinct type IV hysteresis loop in the relative pressure (P/P_0_) range of 0.8–0.9, which is a typical feature of mesoporous structures. This, combined with the pore size distribution data, confirms the presence of abundant mesopores in H-UiO-66. While O-UiO-66 possesses a larger specific surface area, H-UiO-66 exhibits an ultra-high pore volume. These differences in pore structure are expected to influence the ion adsorption and transport properties of the materials, consequently affecting the performance of the UiO-66@CNF separators in batteries [22].

Figure 2e displays the XRD patterns of the composite separators. The characteristic diffraction peaks of UiO-66 at 7.3°, 8.5°, 12.1°, and 18.6° (indexed to the (111), (002), (022), and (004) planes, respectively) and the characteristic peak of CNF at 2θ = 22.5° (corresponding to the (200) plane of cellulose I crystal structure, PDF#03-0289) are preserved in the UiO-66@CNF separators. No impurity peaks are detected within the detection limit of the instrument, confirming the successful embedding of UiO-66 into the CNF gel matrix while maintaining its intact crystal structure. However, significant differences are observed in the cellulose (200) peak region around 2θ = 22.5°. For the H-UiO-66@CNF separator, a broad diffraction peak centered at 22.5° is clearly visible, corresponding to the (200) plane of cellulose I crystal structure (PDF#03-0289). The preservation of this peak with relatively high intensity suggests that the smaller H-UiO-66 particles (50 nm) do not significantly disrupt the crystalline domains of the CNF matrix. The particles appear to be primarily adsorbed on the fiber surfaces without substantially altering the bulk cellulose structure, consistent with the SEM observation of surface-decorated morphology. In contrast, the O-UiO-66@CNF separator shows a significantly reduced intensity and slight broadening of the cellulose (200) peak at 22.5°. This change indicates that the larger octahedral O-UiO-66 particles (300 nm) interact more extensively with the CNF matrix, causing disruption of the cellulose crystalline domains. The larger particles physically intercalate between CNFs during the gel formation process, leading to localized strain and disordering of the cellulose crystal structure. This interpretation is supported by the SEM images (Figure 1e,f) showing O-UiO-66 particles embedded within the CNF network rather than merely surface-adsorbed. The reduced crystallinity of cellulose in the O-UiO-66@CNF separator does not compromise the material’s integrity but rather reflects the more intimate integration of MOF particles within the CNF gel matrix, which is beneficial for creating a composite structure with enhanced mechanical stability and uniform ion transport pathways. This difference in XRD patterns provides additional evidence that the two morphologies result in fundamentally different composite structures: surface-decorated CNF for H-UiO-66 versus intimately integrated gel composite for O-UiO-66. No impurity peaks are detected, confirming the successful embedding of UiO-66 into the CNF gel matrix while maintaining its intact crystal structure. This is crucial for ensuring the stability of the material’s properties [23].

Figure 2f shows the FTIR spectra of the separators. The pure CNF separator exhibits significant -OH and -COOH stretching vibration characteristic peaks in the range of 1000–1250 cm^−1^ (corresponding to the characteristic frequency range for -OH and -COOH stretching vibrations in cellulose molecules), which match the typical absorption bands of cellulose molecules [35,36]. Specifically, the broad absorption band around 3340 cm^−1^ corresponds to the O-H stretching vibration of hydroxyl groups in cellulose, while the peak at 2895 cm^−1^ is attributed to C-H stretching vibration. The intense peak at 1025 cm^−1^ is assigned to C-O-C stretching of the pyranose ring skeleton, and the peak at 1315 cm^−1^ corresponds to C-H bending vibration [37]. Comparative analysis indicates that the infrared spectrum of the composite separator retains the characteristic peaks of both CNF and UiO-66, with no new chemical bond signals appearing. The preservation of all characteristic peaks without significant shifts or the emergence of additional peaks confirms that the interaction between UiO-66 and CNF is primarily physical in nature, involving hydrogen bonding and van der Waals interactions rather than chemical bond formation. This physical combination is advantageous as it maintains the intrinsic properties of both materials while ensuring the structural integrity and stability of the composite separator, which is crucial for maintaining the physicochemical performance of the materials under electrochemical operating conditions.

Figure 2g,h show the contact angle comparison of the UiO-66@CNF separators. The contact angle for the O-UiO-66@CNF separator is 21°, and for the H-UiO-66@CNF separator it is 33°, both smaller than that of the pure CNF separator (48°) (Figure S1b). The significant reduction in contact angles indicates that the UiO-66@CNF separators possess superior electrolyte wettability and hydrophilicity, enhancing their interfacial interaction with the electrolyte. This excellent wettability stems from the inherent high hydrophilicity of UiO-66 itself and is also benefited from the uniform hydrophilic surface provided by CNF. The O-UiO-66@CNF separator demonstrates a lower contact angle and better electrolyte wettability compared to the H-UiO-66@CNF separator, which favors faster zinc ion transport within the separator.

**Figure 3:**
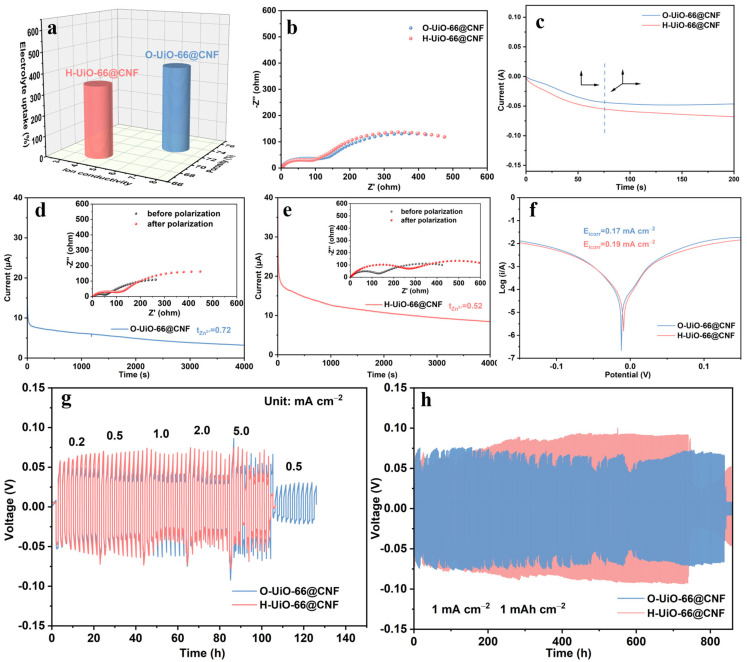
Electrochemical performance of UiO-66@CNF battery separators: (**a**) O-UiO-66@CNF shows higher electrolyte uptake (415.4%) and porosity (73.2%) compared to H-UiO-66@CNF (347.3% and 68.6%), indicating superior electrolyte retention capacity; (**b**) Nyquist plots showing lower charge transfer resistance and smoother ion diffusion for O-UiO-66@CNF separator; (**c**) Chronoamperometry test revealing O-UiO-66@CNF transitions from 2D to stable 3D diffusion within 75 s, effectively suppressing dendrite formation, while pure CNF shows prolonged 2D diffusion exceeding 200 s; (**d**,**e**) Ion migration number tests showing tZn^2+^ of 0.72 for O-UiO-66@CNF versus 0.52 for H-UiO-66@CNF, indicating more efficient Zn^2+^ transport kinetics; (**f**) Tafel polarization curves demonstrating lower corrosion current density for O-UiO-66@CNF (0.17 mA cm^−2^) compared to H-UiO-66@CNF (0.19 mA cm^−2^); (**g**) Rate capability tests showing stable voltage-time profiles and lower polarization for O-UiO-66@CNF across 0.2–5.0 mA·cm^−2^; (**h**) Long-term cycling at 1 mA·cm^−2^ and 1 mAh·cm^−2^ showing O-UiO-66@CNF maintains stable voltage profile over 800 h, while H-UiO-66@CNF shows fluctuations after 750 h due to soft short-circuits from dendrite contact.

Figure 3a compares the porosity and electrolyte uptake of the two separators. The O-UiO-66@CNF and H-UiO-66@CNF composite separators exhibit electrolyte uptake rates of 415.4% and 347.3%, with porosities of 73.2% and 68.6%, respectively. The O-UiO-66@CNF separator demonstrates higher electrolyte uptake and porosity, indicating a superior electrolyte retention capacity. This helps maintain uniform electrolyte distribution, increases zinc ion transport pathways, and facilitates smoother ion transport. This enhancement can be attributed to the porous structure of UiO-66 and its unsaturated metal sites, which may promote electrolyte dissociation, as well as the favorable interaction between UiO-66 and the CNF gel matrix [38]. The interlocking of UiO-66 particles with the fibers likely separates the fibers to some extent, creating additional hydrophilic channels and effectively improving the separator’s liquid absorption capability. Furthermore, as shown in Figure S4c, the bulk resistance of the UiO-66@CNF separator is much lower than that of the CNF separator. This means the UiO-66@CNF separator poses less hindrance to ion conduction, which is more favorable for zinc ion transport through the separator. This property works synergistically with its higher porosity and electrolyte uptake to collectively enhance the ion transport performance of the separator.

**Figure 4:**
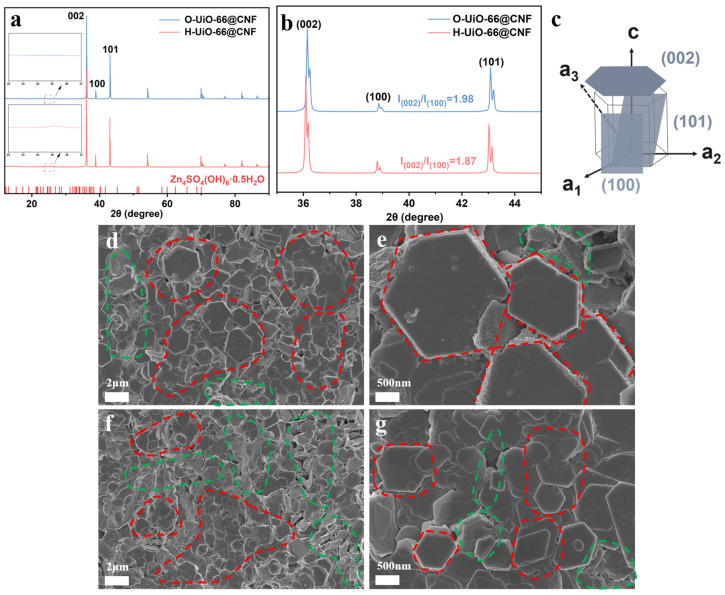
Characterization after 100 cycles at 2 mA cm^−2^ and 1 mAh cm^−2^ with UiO-66@CNF separators: (**a**) XRD patterns; (**b**) Comparison of the relative intensities of the Zn(002) and Zn(100) diffraction peaks; (**c**) Schematic illustration of the crystallographic orientation induced by UiO-66@CNF; Surface SEM images of the cycled anodes with (**d**,**e**) O-UiO-66@CNF separator and (**f**,**g**) H-UiO-66@CNF separator (The red areas indicate horizontally oriented zinc deposition layers, while the green areas represent disordered zinc dendrites growing in vertical and inclined directions).

As observed from Figure 4b and Figure S4d, the battery assembled with the UiO-66@CNF separator exhibits a smaller resistance value in the high-frequency region, indicating lower charge transfer resistance at the electrode/electrolyte interface. In the mid-to-low frequency region, the relatively gentle Warburg diffusion slope of the curve suggests a smooth ion diffusion process and good stability. The low impedance value of the O-UiO-66@CNF separator indicates the best ion conductivity in the electrolyte, which can be attributed to its regular octahedral morphology. During the grafting process with CNF, these particles fill the sparse voids within the CNF, creating more uniform pores. The larger specific surface area facilitates the formation of a homogeneous interface, reduces interfacial impedance for ion conduction, homogenizes the Zn^2+^ flux, and facilitates zinc ion transport.

Furthermore, the zinc ion nucleation processes on the two separators were verified by chronoamperometry tests, as shown in Figure 3c. For the pure CNF separator (Figure S5a), the current-time curve continuously decreases within 200 s, indicating that the zinc ion nucleation undergoes a prolonged two-dimensional diffusion process. Sustained two-dimensional diffusion leads to non-uniform zinc deposition during charge/discharge cycles, potentially resulting in battery short circuits. In contrast, the O-UiO-66@CNF separator transitions from two-dimensional diffusion to a stable three-dimensional diffusion process within 75 s, and exhibits a lower diffusion current. This is likely because the well-dispersed octahedral particles uniformly fill the CNF voids, inducing rapid three-dimensional diffusion and uniform growth of zinc ions through a confinement effect, effectively suppressing zinc dendrite formation [32,39].

Figure 3d,e display the ion transference number test results for the O-UiO-66@CNF and H-UiO-66@CNF separators, respectively. The Zn^2+^ transference number (tZn^2+^) for the O-UiO-66@CNF separator is 0.72, while that for the H-UiO-66@CNF separator is 0.52. Evidently, the UiO-66@CNF separators exhibit significantly higher ion transference numbers than the CNF separator (Figure S5), with the O-UiO-66@CNF separator showing the highest value. Furthermore, both the initial and steady-state currents of the O-UiO-66@CNF separator are noticeably lower than those of the H-UiO-66@CNF separator, indicating lower interfacial charge transfer resistance and more efficient Zn^2+^ transport kinetics. The smaller impedance change of the O-UiO-66@CNF separator before and after polarization further confirms its superior interfacial stability compared to the H-UiO-66@CNF separator. These results demonstrate that the composite separator prepared with O-UiO-66 effectively mitigates the concentration gradient at the separator/zinc anode interface through its uniform pore distribution and optimized interfacial charge distribution. This enables homogeneous regulation of the Zn^2+^ flux, significantly suppresses zinc dendrite growth, and enhances the battery’s cycling stability and electrochemical performance.

Figure 4f and Figure S5b show the Tafel polarization curves comparing the two UiO-66@CNF separators and the CNF separator. Compared to the pure CNF separator, the UiO-66@CNF separators exhibit lower corrosion current densities, indicating an enhanced ability to resist corrosion. Specifically, the H-UiO-66@CNF separator shows a higher corrosion current density (0.19 mA cm^−2^), while the O-UiO-66@CNF separator demonstrates a lower value (0.17 mA cm^−2^). This suggests that the O-UiO-66@CNF separator can more effectively suppress corrosion reactions. This is attributed not only to the high chemical stability of O-UiO-66 itself but also likely to the formation of a more uniform protective layer within the CNF gel matrix, which reduces corrosion sites [40].

As shown in Figure 3g, galvanostatic charge–discharge cycling tests were conducted at current densities ranging from 0.2 to 5.0 mA·cm^−2^. The rate capabilities of both composite separators are superior to that of the pure CNF separator (Figure S5c). The O-UiO-66@CNF separator demonstrates more stable voltage-time profiles and lower polarization voltages across the entire current density range. In contrast, while the H-UiO-66@CNF separator shows acceptable rate performance at low current densities, it fails to adapt to high current densities, evidenced by significantly increased polarization.

In Figure 3h, both the H-UiO-66@CNF and pure CNF separators (Figure S5d) exhibit gradually increasing polarization voltages with prolonged cycling. After 750 h of cycling, the voltage profile of the H-UiO-66@CNF cell shows significant fluctuations, likely induced by soft short-circuits triggered by minor zinc dendrite contact. In comparison, the O-UiO-66@CNF separator exhibits a lower polarization voltage and a much smoother, more stable voltage-time profile. This is attributed to the uniform distribution of the octahedral UiO-66 particles on the surface and within the CNF network, which promotes a homogeneous Zn^2+^ flux and facilitates a uniform zinc nucleation process.

To further investigate the zinc deposition behavior, the cycled zinc anodes and separators were characterized by XRD and SEM. As shown in Figure 4a, which presents the XRD patterns of the zinc anodes using different separators, a peak consistent with the characteristic diffraction of the by-product Zn_4_SO_4_(OH)_4_·0.5H_2_O appears at 25.4° when using the H-UiO-66@CNF separator, albeit with a relatively low intensity, indicating only a small amount of by-product formation. In contrast, no characteristic peaks of by-products are observed on the zinc anode using the O-UiO-66@CNF separator, indicating that the O-UiO-66@CNF separator effectively suppresses the formation of by-products during cycling. This result aligns with the corrosion resistance reflected in the Tafel plot in Figure 3f, namely that the O-UiO-66@CNF separator possesses excellent corrosion resistance. Furthermore, it can be seen from Figure 4b that the intensity ratio of the Zn (002) plane to the Zn (100) plane is high for the zinc anodes corresponding to both separators, with an even higher ratio for the O-UiO-66@CNF case. This indicates a stronger preferential orientation for deposition along the Zn(002) crystallographic plane [41], suggesting that O-UiO-66@CNF can induce zinc deposition preferentially in the horizontal direction (along the 002 direction in Figure 4c), which is conducive to achieving a more uniform zinc deposition layer [42]. This means that both separators can induce horizontal zinc deposition, favoring the formation of a more uniform zinc deposition layer.

**Figure 5:**
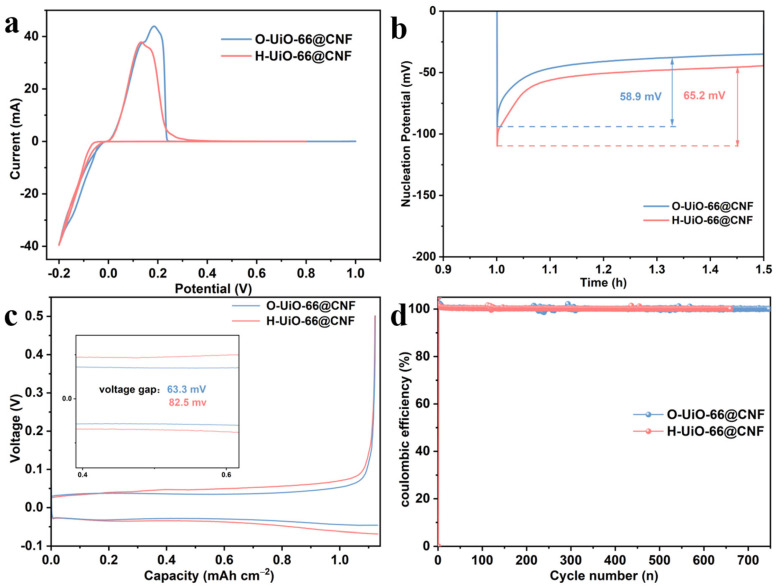
Electrochemical behavior of Zn//Cu half-cells: (**a**) Cyclic voltammetry (CV) curves at 1 mV·s^−1^; (**b**) Nucleation overpotential profiles at 1 mA·cm^−2^ and 1 mAh·cm^−2^; (**c**) Capacity-voltage profiles at 1 mA·cm^−2^ and 1 mAh·cm^−2^; (**d**) Coulombic efficiency at 1 mA·cm^−2^ and 1 mAh·cm^−2^.

Figure 4d–g show surface SEM images at different magnifications of the UiO-66@CNF separators after 100 cycles at 2 mA cm^−2^ and 1 mAh cm^−2^. For the H-UiO-66@CNF separator, while some zinc deposition layers growing along the Zn(002) plane are observed, numerous disordered zinc dendrites with various shapes originating from the Zn(100) and Zn(101) planes are also present. In contrast, the surface of the O-UiO-66@CNF separator exhibits a flat and uniform zinc deposition layer, composed of numerous regularly arranged hexagonal platelets growing horizontally, which are structurally intact and stacked in layers, with only a minimal amount of zinc deposited in inclined directions. This indicates that both separators can homogenize the Zn^2+^ flux and promote horizontal zinc deposition, thus effectively suppressing zinc dendrite growth. The mechanism underlying this preferential deposition can be understood through several factors. First, the well-dispersed O-UiO-66 particles with regular octahedral morphology create a uniform network of micropores (approximately 0.8 nm in diameter, commensurate with hydrated Zn^2+^ ions) that act as ion sieving channels. These channels help homogenize the Zn^2+^ flux and reduce local concentration gradients at the electrode-electrolyte interface. Second, the high specific surface area of O-UiO-66 (as confirmed by BET analysis, Figure 2c) provides abundant nucleation sites, which lowers the nucleation overpotential (Figure 5b) and promotes more uniform zinc deposition. Third, the carboxylate groups on the UiO-66 framework can coordinate with water molecules and Zn^2+^ ions, facilitating the desolvation process and guiding the oriented growth of zinc crystals. The preferential orientation along the Zn(002) plane is particularly advantageous because the (002) plane has the lowest surface energy and is the most thermodynamically stable crystal face for hexagonal close-packed zinc [43,44]. By promoting deposition along this plane, the O-UiO-66@CNF separator encourages the formation of dense, flat zinc layers rather than protrusive dendritic structures that typically grow along the higher-energy (100) and (101) planes. This crystallographic control, combined with the uniform ion flux provided by the well-dispersed MOF particles, effectively suppresses dendrite formation and enables long-term stable cycling. These findings are consistent with the comparative results from the XRD patterns of the post-cycled zinc anodes.

**Figure 6:**
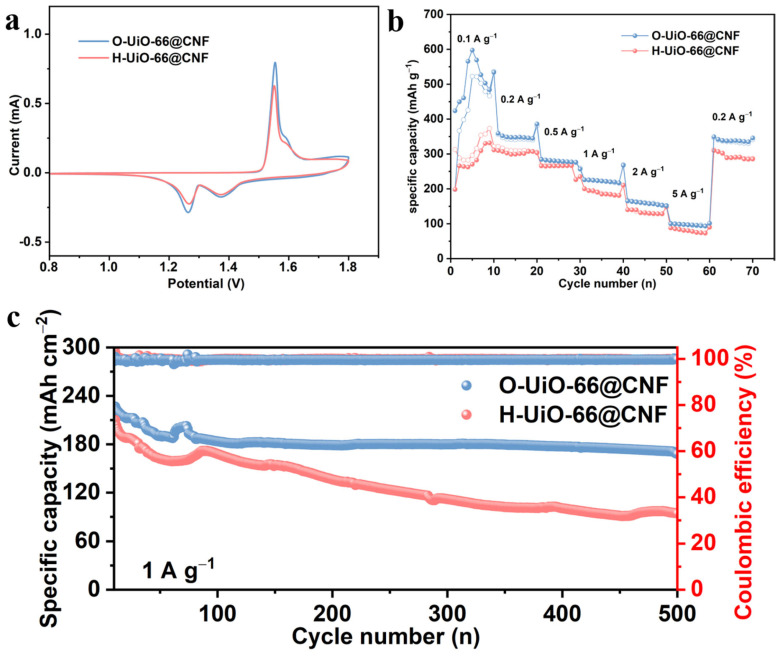
(**a**) CV curves of Zn//MnO_2_ full cells using the two separators at 1 mV·s^−1^; (**b**) Rate capability of the Zn//MnO_2_ full cells with the two separators at different mass densities; (**c**) Long-term cycling performance and Coulombic efficiency of the Zn//MnO_2_ full cells with the two separators at 1 A·g^−1^.

The durability and reversibility of the zinc plating/stripping process with the two separators were further verified by assembling Zn//Cu asymmetric cells. Figure 6a and Figure S6a show the cyclic voltammetry (CV) curves of the Zn//Cu asymmetric cells at a scan rate of 1 mV·s^−1^. The CV curves of both separators show similar shapes and redox peaks. However, the H-UiO-66@CNF separator exhibits a larger potential difference between the oxidation and reduction peaks, indicating a higher degree of polarization during the zinc deposition/dissolution process. In contrast, the O-UiO-66@CNF separator shows the smallest potential difference between the oxidation and reduction peaks, along with the highest peak current density. This suggests a higher capacity and superior reversibility for the zinc deposition/dissolution process.

Figure 6b and Figure S6b present the nucleation overpotential profiles of the Zn//Cu half-cells. The O-UiO-66@CNF separator demonstrates a relatively lower nucleation overpotential, indicating a lower energy barrier for the initial nucleation of zinc ions. This reduces the hindrance to zinc deposition and promotes more uniform zinc plating. This can be primarily attributed to the high specific surface area and uniform particle distribution of the octahedral O-UiO-66, which provides more active sites and leads to a more homogeneous distribution of zinc ions [23].

Figure 5c displays the voltage profiles and corresponding local magnifications of the 10th plating/stripping cycle for the two types of cells. A lower voltage gap is observed for the cell with the O-UiO-66@CNF separator, indicating its superior capability in enhancing the durability and reversibility of the zinc plating/stripping process [41].

Figure 5d shows the Coulombic efficiency curves of the two half-cells tested at 1 mA·cm^−2^ and 1 mAh·cm^−2^. Both half-cells assembled with the composite separators exhibited long cycle life, with an average Coulombic efficiency exceeding 99%.

Zn//MnO_2_ full cells were assembled to evaluate the practical applicability of the separators. As shown in Figure S7, the diffraction peaks and crystal planes of the α-MnO_2_ synthesized via the hydrothermal method correspond well with the standard PDF#44-0141 card, confirming the successful synthesis of α-MnO_2_.

Figure 6a displays the cyclic voltammetry (CV) curves of the full cells employing the two separators at a scan rate of 1 mV·s^−1^. The CV curves of the full cells with both separators exhibit similar shapes, indicating that the separator morphology does not alter the fundamental reaction mechanism of the battery. In comparison, the cell with the O-UiO-66@CNF separator shows increased peak currents for both the oxidation and reduction peaks, suggesting higher electrochemical activity. This difference is likely due to the distinct influences of the different UiO-66 morphologies on the electrochemical behavior during the zinc ion reduction process. The H-UiO-66 with its hierarchical porous structure exhibits poorer dispersion compared to the octahedral structure of O-UiO-66, making it prone to surface aggregation effects. This weakens the interfacial interaction between the electrolyte and the separator. Furthermore, the hierarchical structure might lead to locally elevated zinc ion concentrations, thereby affecting the zinc ion reduction behavior. These effects manifest in the CV curves as differences in the reduction peak width and the potential difference between the peaks.

Figure 6b presents the rate capability of the cells with different separators at various mass loadings. As the mass loading increased, the discharge specific capacity decreased for both full cells. The full cell with the O-UiO-66@CNF separator exhibited a lower specific capacity at low mass loadings, but demonstrated a higher specific capacity than the cell with the H-UiO-66@CNF separator at high mass loadings, indicating its better tolerance to varying mass loadings. When the current density was returned to 0.2 A g^−1^, the O-UiO-66@CNF cell showed a smaller capacity loss compared to the H-UiO-66@CNF cell, reflecting its superior capacity recovery.

Figure 6c shows the long-term cycling performance and Coulombic efficiency of the cells at 1 A g^−1^. Both cells exhibited similar initial discharge specific capacities (226.7 mAh g^−1^ for O-UiO-66@CNF and 210.4 mAh g^−1^ for H-UiO-66@CNF). However, the discharge specific capacity of the H-UiO-66@CNF cell continuously decreased throughout the cycling test, dropping to 94.7 mAh g^−1^ after 500 cycles with a capacity retention of only 45.0%. In contrast, the discharge specific capacity of the O-UiO-66@CNF cell stabilized after 100 cycles, with a very low capacity decay rate of only 0.019% per cycle, while the Coulombic efficiency consistently remained near 100%. The higher discharge specific capacity and capacity retention demonstrate the excellent long-term cycling stability and electrochemical reversibility of the O-UiO-66@CNF separator.

**Scheme 1:**
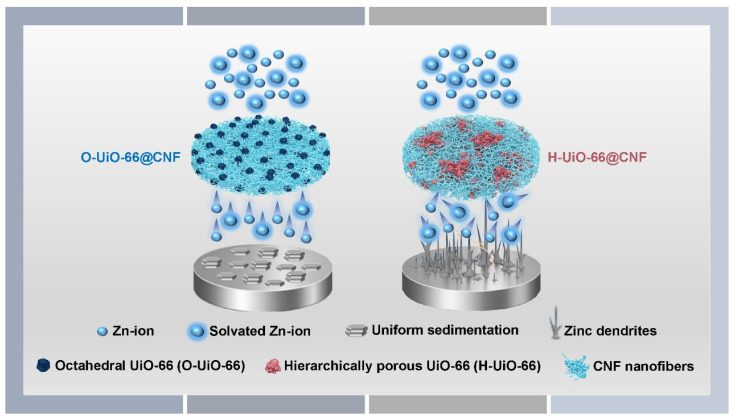
Schematic illustration of zinc deposition on UiO-66@CNF battery separators with different morphologies.

In summary, the regular octahedral morphology of O-UiO-66 facilitates its uniform dispersion within the CNF network, effectively minimizing particle agglomeration. In contrast, the irregular hierarchical porous morphology of H-UiO-66 is prone to particle aggregation. As illustrated in Scheme 1, O-UiO-66 particles are evenly distributed on the surface and within the CNFs, forming continuous and ordered ion transport channels. Their high specific surface area provides abundant sites for zinc ion deposition, while the regularly arranged pores facilitate the desolvation and uniform flux of zinc ions. This reduces the local current density at the zinc deposition interface, enabling uniform zinc plating. Furthermore, the homogeneous distribution of O-UiO-66 enhances the interfacial compatibility with CNF, creating a more robust separator structure that effectively suppresses zinc dendrite penetration and enhances the cycling stability of the full cell. While direct mechanical characterization was not performed in this study, the uniform embedding of O-UiO-66 particles within the CNF network, as evidenced by SEM analysis (Figure 1e,f), suggests improved structural stability compared to the agglomerated H-UiO-66 morphology (Figure 1g,h). Future work will include comprehensive mechanical testing to quantify the tensile strength, flexibility, and puncture resistance of these composite separators. Conversely, the agglomeration of H-UiO-66 particles disrupts the homogeneity of the separator’s pore structure, not only hindering efficient ion transport but also exacerbating uneven local current density distribution. This induces non-uniform zinc dendrite growth and side reactions, ultimately leading to battery performance degradation.

## 3. Conclusions

This study successfully demonstrates a gel-based strategy for designing high-performance separators for aqueous zinc-ion batteries (AZIBs) by incorporating morphology-controlled UiO-66 into a cellulose nanofiber (CNF) gel matrix. The central finding is that the regular octahedral morphology of UiO-66 (O-UiO-66) enables its uniform dispersion within the CNF hydrogel network, leading to the formation of a well-defined, continuous three-dimensional ion transport channel. In contrast, the hierarchically porous UiO-66 (H-UiO-66) tends to agglomerate, disrupting the homogeneity of the gel structure.

The resultant O-UiO-66/CNF gel-derived separator exhibits exceptional material properties, including outstanding hydrophilicity (contact angle of 21°), high porosity (73.2%), and superior electrolyte uptake, which collectively contribute to a significantly enhanced zinc ion migration number (0.72). Electrochemical assessments confirm that the homogeneous gel structure of the O-UiO-66/CNF separator effectively guides uniform Zn^2+^ flux and induces horizontal zinc deposition along the (002) plane, thereby significantly suppressing dendrite growth and parasitic reactions. Consequently, Zn//Zn symmetric cells assembled with this gel-based separator achieve an extended cycle life of over 800 h at 1 mA cm^−2^, and the Zn//MnO_2_ full cell maintains 98.1% capacity retention after 100 cycles at 1 A g^−1^.

To contextualize the significance of our findings, it is instructive to compare our O-UiO-66@CNF separator with the recently reported UiO-66/bacterial cellulose separator by Hu et al. [16]. Table 2 provides a comprehensive comparison of key performance metrics. Our O-UiO-66@CNF separator demonstrates superior performance in several critical aspects: (1) higher zinc ion transference number (0.72 vs. 0.65), indicating more efficient Zn^2+^ transport; (2) longer cycle life in symmetric cells (800+ h vs. 600 h at 1 mA cm^−2^); (3) better capacity retention in full cells (98.1% after 100 cycles vs. 92.3% after 80 cycles). These improvements can be attributed to the unique combination of CNF gel matrix and morphology-controlled UiO-66 octahedral particles, which creates a more uniform and efficient ion transport network. Furthermore, our work provides deeper mechanistic insights into the role of MOF morphology in governing ion transport and zinc deposition behavior, which was not addressed in the previous study. The systematic investigation of different morphologies and their impact on gel structure represents a significant advancement beyond the previous work’s demonstration of feasibility.

To evaluate the practical significance of our O-UiO-66@CNF separator, it is essential to compare its performance with other recently reported separators for AZIBs. Table 3 summarizes the key performance metrics of various state-of-the-art separators, including commercial glass fiber (GF), polymer-based separators, and other MOF/COF-modified separators.

As shown in the comparison table, our O-UiO-66@CNF separator demonstrates superior performance across multiple key metrics. The zinc ion transference number of 0.72 is among the highest reported for MOF-based separators, indicating highly efficient ion transport. The symmetric cell cycle life exceeding 800 h represents a significant improvement over most reported separators, demonstrating excellent dendrite suppression capability. The full cell capacity retention of 98.1% after 100 cycles is comparable to or better than the best-performing separators reported to date.

The advantages of our separator can be attributed to several factors: (1) The regular octahedral morphology of UiO-66 provides uniform pore distribution and high specific surface area. (2) The gel-derived CNF matrix ensures excellent electrolyte wettability and structural stability. (3) The synergistic combination of MOF and CNF creates an optimized ion transport network. However, we acknowledge certain limitations: (1) The synthesis process involves multiple steps, which may increase production costs compared to simple mixing approaches. (2) The thickness of our separator (approximately 150 μm) is greater than some commercial separators, potentially affecting volumetric energy density. (3) Long-term cycling data (>1000 cycles) would be needed to fully establish the practical viability for commercial applications. Future work will focus on optimizing the separator thickness, simplifying the fabrication process, and conducting extended cycling tests to address these limitations.

In summary, this work underscores the critical impact of MOF crystal morphology on the microstructure and performance of CNF-based gel composites. The strategy of constructing a uniform gel network through morphology control provides a novel and efficient pathway for developing advanced functional materials for energy storage devices. The insights gained from this study on the structure–property relationship in gel composites are expected to inspire further exploration in the design of gel-based materials for a wide range of applications.

## 4. Experimental Materials and Methods

### 4.1. Materials

Cellulose nanofiber (CNF) aqueous dispersion (solid content 1.8%, purchased from Zhejiang Jinjiahao Green Nano Material Co., Ltd., Quzhou, China), Zirconium(IV) propoxide solution (C_12_H_28_O_4_Zr, 70 wt.% in 1-propanol, purchased from Shanghai Aladdin Biochemical Technology Co., Ltd., Shanghai, China), Terephthalic acid (PTA, C_8_H_6_O_4_, 99%, purchased from Shanghai Aladdin Biochemical Technology Co., Ltd., Shanghai, China), Acetic acid (HAc, CH_3_COOH, AR, 99.5%, purchased from Shanghai Aladdin Biochemical Technology Co., Ltd., Shanghai, China), *N*,*N*-Dimethylformamide (DMF, C3H7NO, AR, 99.5%, purchased from Shanghai Aladdin Biochemical Technology Co., Ltd., Shanghai, China), Manganese sulfate monohydrate (MnSO_4_·H_2_O, AR, 99%, purchased from Shanghai Aladdin Biochemical Technology Co., Ltd., Shanghai, China), Potassium permanganate (KMnO_4_, ≥99.0%, purchased from Shanghai Aladdin Biochemical Technology Co., Ltd., Shanghai, China), 1-Methyl-2-pyrrolidinone (NMP, C_5_H_9_NO, AR, >99.0% (GC), purchased from Shanghai Aladdin Biochemical Technology Co., Ltd., Shanghai, China), Super P carbon black (purchased from Future (Jilin) Materials Technology Co., Ltd., Jilin, China), Poly(vinylidene fluoride) (PVDF, purchased from Future (Jilin) Materials Technology Co., Ltd., Jilin, China). All other chemical reagents were of analytical grade and used without further purification.

The cellulose nanofiber (CNF) aqueous dispersion used in this study was purchased from Zhejiang Jinjiahao Green Nano Material Co., Ltd. This CNF is derived from plant cellulose through mechanical and chemical treatment processes, resulting in nanofibers with diameters of 10–50 nm and lengths of several micrometers. Unlike bacterial cellulose which consists of pure cellulose I crystal structure with high crystallinity (>90%), plant-derived CNF typically contains both cellulose I and amorphous regions, providing different surface chemistry and mechanical properties that influence its gel formation behavior and interaction with MOF particles.

#### Scientific Rationale for Morphology Selection

UiO-66, a zirconium-based metal–organic framework, is known for its exceptional structural tunability, with various morphologies reported in the literature including octahedral [29], cubic [18], rod-like [20], plate-like [38], and hierarchical porous structures [51]. Each morphology exhibits distinct physicochemical properties that can significantly influence its performance in energy storage applications. Octahedral UiO-66, with its well-defined facets and high specific surface area, has been shown to provide abundant active sites and uniform pore distribution, making it particularly suitable for ion transport applications [22]. The octahedral morphology is thermodynamically favored under conditions of moderate supersaturation and controlled nucleation rates, which can be achieved through precise modulation of reactant concentrations and solvent environment [52].

Hierarchical porous UiO-66, characterized by the presence of both micropores and mesopores, offers enhanced mass transport properties due to its bimodal pore size distribution [53]. However, the synthesis of hierarchical structures often involves rapid nucleation and growth under high supersaturation conditions, which can lead to incomplete crystallization and morphological defects [54]. The presence of mesopores, while beneficial for bulk transport, may compromise the molecular sieving capability essential for uniform ion flux regulation.

In this study, we selected octahedral and hierarchical porous morphologies for comparison based on the following scientific considerations: (1) Octahedral morphology represents the most thermodynamically stable and well-crystallized form of UiO-66, providing a baseline for understanding structure–property relationships. (2) Hierarchical porous morphology represents a kinetically controlled structure with enhanced porosity but potentially compromised crystallinity. (3) These two morphologies exemplify the fundamental trade-off between crystallinity/pore regularity and porosity/mass transport in MOF-based separators. By systematically comparing these representative structures, we aim to establish design principles for optimizing MOF morphology in gel-based separators for AZIBs.

The synthesis protocol was designed based on established principles of MOF crystallization [55]. The formation of octahedral morphology (O-UiO-66) was achieved through controlled nucleation at moderate concentrations, allowing for facet-selective growth and development of well-defined crystal habits. The hierarchical porous morphology (H-UiO-66) was obtained by diluting the reaction system fivefold, which increases the supersaturation ratio and promotes rapid, uncontrolled nucleation, leading to smaller crystallites with morphological defects and interparticle mesopores. This deliberate design allows for direct comparison of how crystallinity, particle size, and pore structure affect separator performance, providing insights that can guide future development of optimized MOF morphologies for energy storage applications.

### 4.2. Material Preparation

#### 4.2.1. Synthesis of UiO-66 with Different Morphologies

Two types of UiO-66 with distinct morphologies were synthesized using a two-step solvothermal method. The specific preparation procedure is as follows: First, 0.600 mmol of zirconium(IV) propoxide, used as the metal precursor, was mixed with 21.612 mL of DMF and 12.350 mL of HAc in two separate, identical reaction vessels. The reaction systems were placed in an oven at 130 °C for 3 h, then allowed to cool naturally to room temperature. The resulting mixtures were transferred to Beakers A and B for subsequent steps.

The second synthesis step was then conducted: 0.900 mmol of terephthalic acid (H_2_BDC) was added as the organic ligand to Beaker A. Simultaneously, the reaction system in Beaker B was diluted fivefold by adding 170 mL of DMF solvent, followed by the addition of an equal amount of H_2_BDC (0.900 mmol). Both mixtures were subjected to ultrasonication for 30 min for homogenization. The two reaction systems were then stirred continuously at room temperature for 24 h to ensure complete reaction.

**Figure 7:**
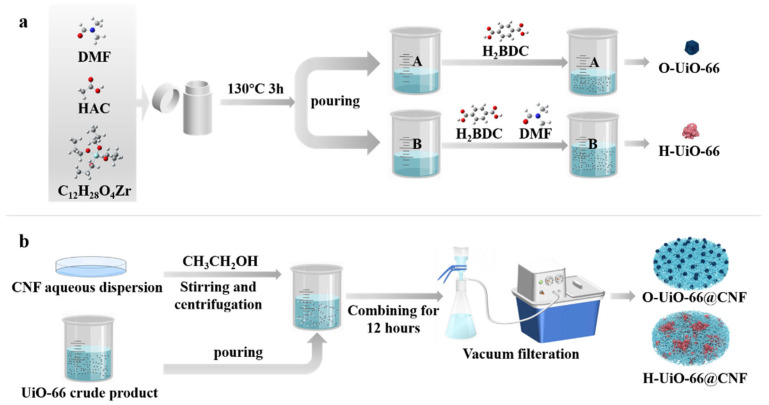
(**a**) Schematic diagram of the synthesis process for UiO-66 with different morphologies; (**b**) Flowchart illustrating the fabrication process of UiO-66@CNF battery separators with different microstructures.

Solid–liquid separation was performed via centrifugation to collect the precipitates. To thoroughly remove reaction residues, the products were washed and purified three times with DMF. Finally, the purified samples were placed in a vacuum drying oven and dried at 80 °C for 12 h to obtain white powdered UiO-66 samples. The product from Beaker A was designated O-UiO-66, and the product from Beaker B was designated H-UiO-66. A schematic diagram of the procedure is shown in Figure 7a.

#### 4.2.2. Fabrication of UiO-66@CNF Battery Separators

First, 12 g of the CNF aqueous dispersion was dispersed in ethanol and left overnight. The resulting mixture was then centrifuged and washed three times before being set aside for use. Subsequently, the two types of UiO-66 with different morphologies were separately dispersed in anhydrous ethanol. These dispersions were mixed with the prepared CNF suspension and stirred. The mixtures were homogenized for 30 min in an ultrasonic cleaner, followed by co-mixing and grafting for 12 h. After this reaction period, the products were centrifuged and washed three times. The resulting materials were then redispersed in anhydrous ethanol and left overnight. Subsequently, wet films were formed via vacuum filtration (details shown in Figure 7b) using a funnel setup with a nylon 66 (N66) microporous membrane filter (pore size 0.45 μm, diameter 90 mm, purchased from Haiyan Xindongfang Plastic Technology Co., Ltd. Jiaxing, China) as the filtration substrate. The UiO-66@CNF suspension was poured onto the filter membrane and vacuum was applied gradually to ensure uniform deposition and prevent particle segregation. The filtration process was continued until no visible liquid passed through the funnel, typically requiring 5–10 min for complete filtration of the 50 mL suspension (the filtration duration was shortened owing to the larger membrane area and larger pore size compared with small-sized, fine-pore membranes).

After filtration, the formed wet film (approximately 2 mm thick) remained adhered to the filter membrane. To separate the film from the membrane, the filter assembly was carefully inverted and the edge of the film was gently lifted using a stainless-steel spatula. The flexible nature of the wet gel film allowed it to be peeled away from the membrane without tearing or leaving residue. The separated wet film was then placed between two sheets of smooth PTFE plates to maintain flat geometry during drying. The assembly was transferred to an oven and dried at 80 °C for 4 h under ambient pressure to remove residual ethanol and water, resulting in a self-standing separator membrane with final thickness of approximately 150 μm. The dried separator could be easily handled and punched into circular disks (17 mm diameter) for battery assembly without cracking or delamination.

#### 4.2.3. Preparation of MnO_2_ Cathode Sheets

First, 0.3803 g of MnSO_4_·H_2_O (2.25 mmol) was weighed and dissolved in 15 mL of deionized water with stirring until completely dissolved, resulting in a homogeneous MnSO_4_ solution. Separately, 0.237 g of KMnO_4_ (1.5 mmol) was weighed and dissolved in 15 mL of deionized water with stirring to form a homogeneous KMnO_4_ solution. The KMnO_4_ solution was then added dropwise into the MnSO_4_ solution, generating a dark brown α-MnO_2_ precipitate. After magnetically stirring the mixture at room temperature for 2 h, it was transferred to a 100 mL stainless-steel autoclave for a hydrothermal reaction at 160 °C for 12 h. Once the autoclave cooled to room temperature, the mixture was centrifuged, and the α-MnO_2_ precipitate was washed three times with deionized water, dried at 80 °C for 12 h, and ground to obtain α-MnO_2_ powder.

The resulting α-MnO_2_ powder was used as the cathode active material. Using NMP as the solvent, a uniform slurry was prepared by thoroughly grinding a mixture of α-MnO_2_, Super P conductive carbon black, and PVDF binder in a mass ratio of 7:2:1. The slurry was then doctor-bladed onto graphite foil and dried in a vacuum oven at 80 °C for 12 h. After cooling to room temperature, the coated foil was punched into 12 mm × 12 mm cathode sheets for battery assembly.

### 4.3. Characterization and Testing Methods

#### 4.3.1. Materials Characterization

The microscopic morphology of the powder samples and separator surfaces was observed using a ZEISS Sigma 300 scanning electron microscope (SEM) (Carl Zeiss AG, Oberkochen, Germany) at different magnifications with an acceleration voltage of 3–5 kV. The crystallinity and crystal structure of the UiO-66 powders, separators, and MnO_2_ cathode active material were characterized by a Bruker D8 Advance X-ray diffractometer (XRD) (Bruker, Karlsruhe, Germany) using Cu Kα radiation (λ = 1.54182 Å). The phase of MnO_2_ was identified with reference to the standard PDF#44-0141 card. Fourier transform infrared (FTIR) spectra of the CNF separator, UiO-66 powder, and UiO-66@CNF composite separator were recorded on a Nicolet iS50 (Thermo Fisher Scientific, Waltham, MA, USA) to analyze the chemical functional groups and bonding information. The specific surface area and pore structure of UiO-66 were determined by nitrogen adsorption–desorption measurements using a Quantachrome Autosorb IQ MP automated surface area and porosity analyzer (Quantachrome Instruments Inc., Boynton Beach, FL, USA). The hydrophilicity and electrolyte wettability of the separators were evaluated by contact angle measurements performed on a CA-100B contact angle goniometer (Shanghai Yingnuo Instrument Co., Ltd., Shanghai, China).

#### 4.3.2. Battery Testing

CR2025 coin-type cells were assembled in air at room temperature. All components were pre-cleaned by ultrasonication in anhydrous ethanol and dried in an oven at 80 °C. The assembly sequence (from bottom to top) was negative case, spacer, spring, negative electrode, electrolyte, separator, positive electrode, and positive case.

Symmetric Cell Tests: Cells were assembled using a 2.0 mol·L^−1^ ZnSO_4_ aqueous solution as the electrolyte, zinc foil as both symmetric electrodes, and a separator with a diameter of 17 mm. Tests included ionic conductivity; electrochemical impedance spectroscopy (EIS); Zn^2+^ transference number (tZn^2+^); chronoamperometry (i-t); cyclic voltammetry (CV); activation energy; as well as the rate capability and cycling stability of the symmetric cells.

Half-Cell Tests: Cells were assembled using a 2.0 mol·L^−1^ ZnSO_4_ electrolyte with copper foil (as the working electrode for Zn deposition/stripping) and zinc foil (as the counter/reference electrode). Tests performed included Tafel polarization curves; cyclic voltammetry (CV) of the half-cells; nucleation overpotential (tested by linear sweep voltammetry); capacity-voltage curves; and coulombic efficiency.

Full-Cell Tests: A mixed solution of 2.0 mol·L^−1^ ZnSO_4_ and 0.2 mol·L^−1^ MnSO_4_ was used as the electrolyte, with MnO_2_ sheets as the cathode and zinc foil as the anode. The full cells were tested for cyclic voltammetry (CV), rate capability, and long-term cycling stability.

### 4.4. Gel Formation Mechanism and Stability

The designation of our material as a ‘gel-derived’ separator requires clarification of the gel formation mechanism. Cellulose nanofibers (CNFs) can form physical hydrogels through multiple mechanisms including hydrogen bonding, entanglement, and hydrophobic interactions [56,57]. In our system, the CNF aqueous dispersion (1.8% solid content) initially exists as a stable colloidal suspension. During the fabrication process, solvent exchange from water to ethanol followed by vacuum filtration induces the formation of a CNF network through physical crosslinking.

The gel formation in our system relies on three primary mechanisms: (1) Hydrogen bonding between CNF, facilitated by the numerous hydroxyl groups on the cellulose surface. (2) Physical entanglement of the high-aspect-ratio nanofibers (diameter 10–50 nm, length several micrometers). (3) Capillary forces during the drying process that bring nanofibers into close contact. The resulting material exhibits gel-like properties including high water absorption capacity (electrolyte uptake of 415.4% for O-UiO-66@CNF) and a three-dimensional network structure, as confirmed by SEM analysis (Figure 1e–h).

To verify the gel stability, we conducted additional experiments where the separators were immersed in 2.0 M ZnSO_4_ electrolyte for extended periods. The O-UiO-66@CNF separator maintained its structural integrity without disintegration over prolonged periods, confirming the stability of the physical gel network under battery operating conditions. The uniform distribution of UiO-66 particles within this gel matrix (Figure 1e,f) indicates that the MOF particles are physically entrapped within the CNF network, creating a composite gel structure with enhanced functionality.

We acknowledge that our material represents a physical gel rather than a chemically crosslinked hydrogel. The term ‘gel-derived’ in our manuscript refers to the processing route involving gel formation through physical crosslinking, followed by controlled drying to obtain the final separator membrane. This approach differs from traditional separator fabrication methods and leverages the unique properties of CNF gels to create well-defined ion transport channels.

## Figures and Tables

**Table 1:** Basic information on pore structure of UiO-66 samples.

Samples	*S*_BET_/ (m^2^ g^−1^)	*S*_Langmuir_/ (m^2^ g^−1^)	*V*_pore_/ (cm^3^ g^−1^)
O-UiO-66	1589.4	1847.8	0.67
H-UiO-66	1066.8	1605.1	1.46

**Table 2:** Performance comparison of UiO-66/cellulose composite separators for AZIBs.

Performance Metric	This Work (O-UiO-66@CNF)	Hu et al. [16] (UiO-66/BC)	Improvement
Ion Transference Number	0.72	0.65	+10.8%
Symmetric Cell Cycle Life	800+ h	600 h	+33.3%
Full Cell Capacity Retention (100 cycles)	98.1%	92.3% (80 cycles)	+6.3%
Contact Angle	21°	28°	Better wettability
Porosity	73.2%	68.5%	+6.9%

**Table 3:** Performance comparison of various separators for aqueous zinc-ion batteries.

Separator Type	Ion Transference Number (t(Zn^2+^))	Symmetric Cell Cycle Life	Full Cell Performance	Ref.
Commercial Glass Fiber (GF)	~0.38	~100 h at 1 mA cm^−2^, 1 mAh cm^−2^	Zn//MnO_2_, 81% capacity retention after 1000 cycles at 1 A g^−1^	[45]
Celgard 2500 (PP)	~0.26	Rapid short circuit (<20 h) at 0.5 mA cm^−2^	Poor rate performance due to high polarization	[46]
Cellulose Filter Paper	~0.40	~300 h at 1 mA cm^−2^, 1 mAh cm^−2^	Zn//NH_4_V_4_O_10_, 82% retention after 800 cycles at 5 A g^−1^	[47]
UiO-66@GF	0.67	1650 h at 1 mA cm^−2^ (39 mV polarization)	85% retention after 1000 cycles at 1.0 A g^−1^ (230.8 mAh g^−1^ at 0.1 A g^−1^)	[48]
MOF-based: ZIF-8 coated PP	~0.65	>800 h at 1 mA cm^−2^, 1 mAh cm^−2^	Zn//AC hybrid capacitor, 92% retention after 20,000 cycles	[49]
Cellulose/ZrO_2_ (ZC)	0.69	2000 h at 0.5 mA cm^−2^	Enhanced cyclability in Zn//MnO_2_	[50]
This work: O-UiO-66@CNF	0.72	>800 h at 1 mA cm^−2^, 1 mAh cm^−2^	98.1% retention rate (100 cycles)	-

## Data Availability

All relevant data are within the paper.

## Supplementary Materials

The following supporting information can be downloaded at: https://www.mdpi.com/article/10.3390/gels12010075/s1, Figure S1. SEM images of UiO-66@CNF separators at different magnifications corresponding, (a,b) O-UiO-66@CNF separator, (c,d) H-UiO-66@CNF separator. Figure S2. Histogram of particle size distribution based on SEM data (a) O-UiO-66; (b) H-UiO-66. Figure S3. (a) Zeta potential of UiO-66 samples; separator (b) Surface SEM image of CNF separator; (c) Contact angle of CNF separator. Figure S4. (a) Ion conductivity of CNF separator; (b) Porosity and electrolyte uptake of CNF separator; (c) Nyquist plot of EIS of CNF separator. Figure S5. (a) CA curve at −150 mV overpotential of CNF separator; (b) The plot of Tafel analysis of CNF separator; (c) Zn^2+^ ion transfer number of CNF separator at 10 mV polarization voltage (the insets illustrate the changes in EIS before and after polarization); (d) Rate performance of CNF separator of Zn//Zn symmetric cells at various current densities; (e) Cycling performance of CNF separator of Zn//Zn symmetric cells at a current density of 1 mA·cm^−2^ with an areal capacity of 1 mAh·cm^−2^. Figure S6. Electrochemical behaviors of Zn//Cu asymmetric cell with CNF separator; (a) CV curve at a scan rate of 1 mV·s^−1^; (b) Nucleation overpotential curve at a current density of 1 mA·cm^−2^ with an areal capacity of 1 mAh·cm^−2^; (c) Voltage-capacity profiles at a current density of 1 mA·cm^−2^ with an areal capacity of 1 mAh·cm^−2^ (the inset displays an amplified voltage gap from 0.07 to 0.2 mAh·cm^−2^); (d) Coulombic efficiency at a current density of 1 mA·cm^−2^ with an areal capacity of 1 mAh·cm^−2^. Figure S7. XRD pattern of α-MnO_2_.
