# Supplementary material for: Impact of Gel-Derived Morphology-Controlled UiO-66/Cellulose Nanofiber Composite Separators on the Performance of Aqueous Zinc-Ion Batteries
Source: Gels. 2026 Jan 15;12(1):75. doi: 10.3390/gels12010075 (PMC12841272; doi:10.3390/gels12010075)
Supplement: Supplementary file 1 [file gels-12-00075-s001.zip › gels-4041809-supplementary.pdf]

Impact of Gel-Derived Morphology-Controlled UiO-66/Cellulose Nanofiber Composite Separators on the Per-formance of Aqueous Zinc-Ion Batteries

Tiao Zhao[1,4*], Jiangrong Yu[1,4], Shilin Peng[1,4], Yan Wu[1], Tianhang Wang[1], Zhuoheng Li[1], Ling Shen[2], Christoph Janiak[3], Yi Chen[1*]

[1] School of Materials Science and Engineering, Hunan University of Technology, Zhuzhou, Hunan 412007, China

[2] Center for Materials Research and Analysis, Wuhan University of Technology, Wuhan 430070, China

[3] Institut für Anorganische Chemie und Strukturchemie, Universität Düsseldorf, Universitätsstr. 1,

D-40225 Düsseldorf, Germany

[4] Moxi Advanced Materials Institute (MAMI), Hunan Xuanjing Hechuang New Materials Technology Co., Ltd., Zhuzhou, Hunan 412007, China

* Corresponding authors. E-mail addresses: tian_zhao@hut.edu.cn (T. Zhao), chenyi@hut.edu.cn (Y.Chen)

Abstract: Zinc dendrite growth and side reactions have been shown to be significant factors hindering progress in the field of aqueous zinc-ion batteries (AZIBs). This study proposes a methodology for enhancing the performance of separators through the regulation of the crystal morphology of metal–organic

framework material (UiO-66). The findings demonstrate that, in comparison to hierarchical pore structures (H-UiO-66), octahedral morphology (O-UiO-66) disperses more uniformly within the cellulose nanofiber (CNF) matrix, thereby forming well-defined ion transport channels. The fabricated O-UiO-66@CNF separator exhibits outstanding hydrophilicity (contact angle 21°), high porosity (73.2%), and significantly enhanced zinc ion migration number (0.72). Electrochemical testing demonstrates that this separator effectively guides uniform zinc deposition while significantly suppressing dendrite growth. Zn/Zn symmetric cells, based on the O-UiO-66@CNF separator, have demonstrated a cycle life in excess of 800 hours at 1 mA cm^{-2}, thus indicating a high level of stability. The Zn/MnO_2 full cell exhibited an impressive capacity retention of 98.1% after 100 cycles at 1 A g^{-1}, underscoring its remarkable durability. This work reveals a structure-performance relationship between MOF morphology and separator properties, providing new insights for designing high-performance AZIBs.

Keywords: Aqueous Zinc-ion Batteries; UiO-66; Cellulose Nanofibers; Battery Separator; Suppressing Zinc Dendrites

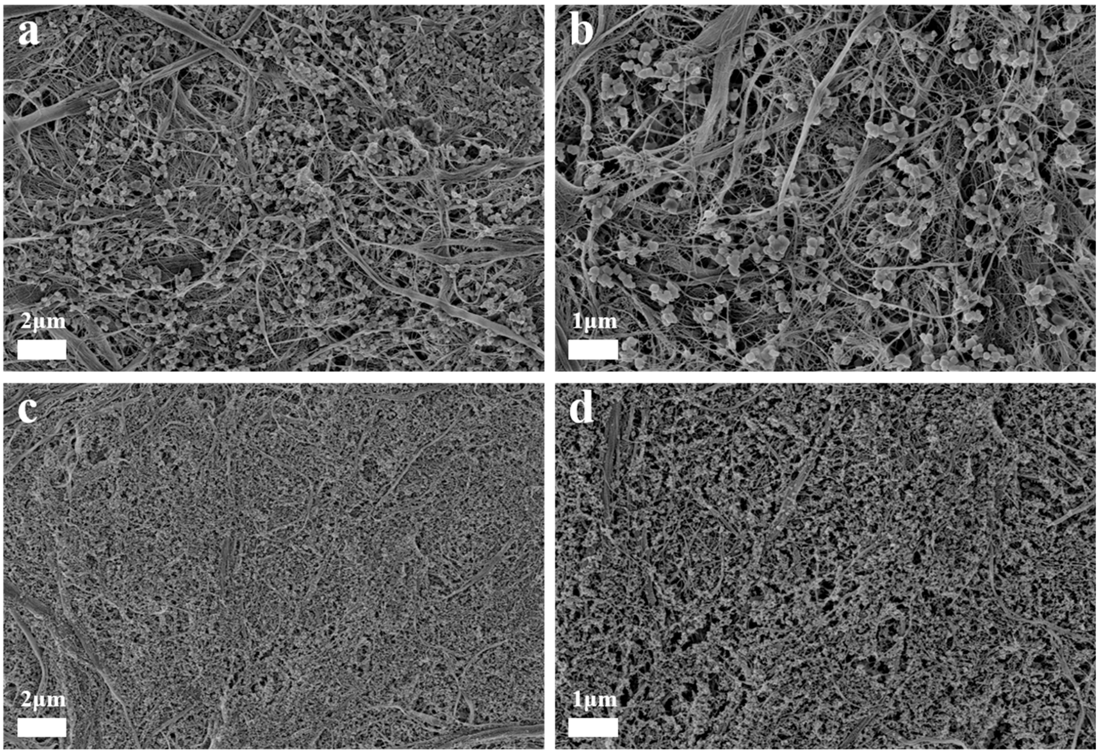

Figure S1. SEM images of UiO-66@CNF separators at different magnifications corresponding, (a)-(b) O-UiO-66@CNF separator, (c)-(d) H-UiO-66@CNF separator

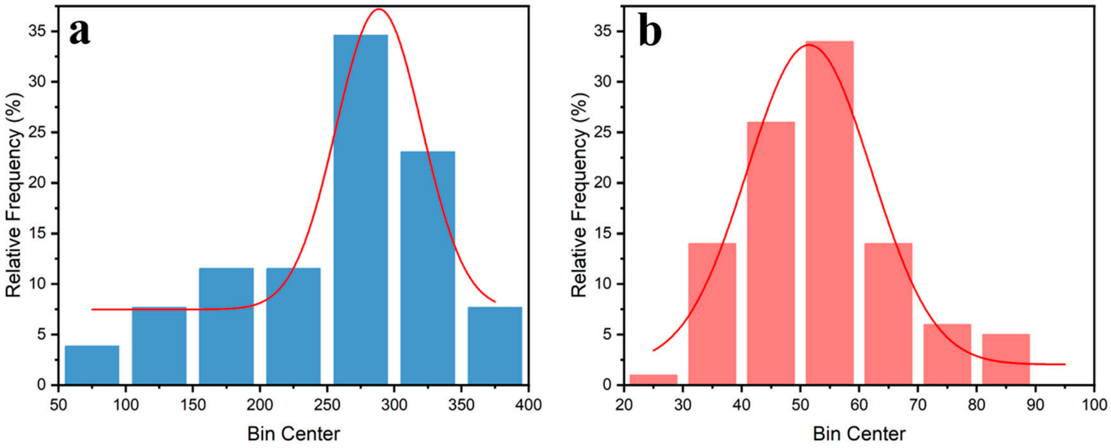

Figure S2. Histogram of particle size distribution based on SEM data (a) O-UiO-66; (b) H-UiO-66

Particle size distribution histograms for O-UiO-66 and H-UiO-66 based on measurements of 200 particles from SEM images. The O-UiO-66 shows a narrow size distribution centered at 298 ± 45 nm, while H-UiO-66 exhibits a broader distribution centered at 52 ± 18 nm.

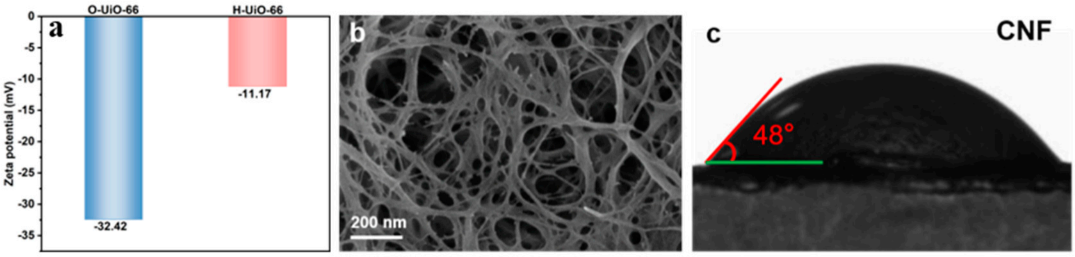

Figure S3. (a) Zeta potential of UiO-66 samples; separator ((b) Surface SEM image of CNF separator; (c) Contact angle of CNF separator.

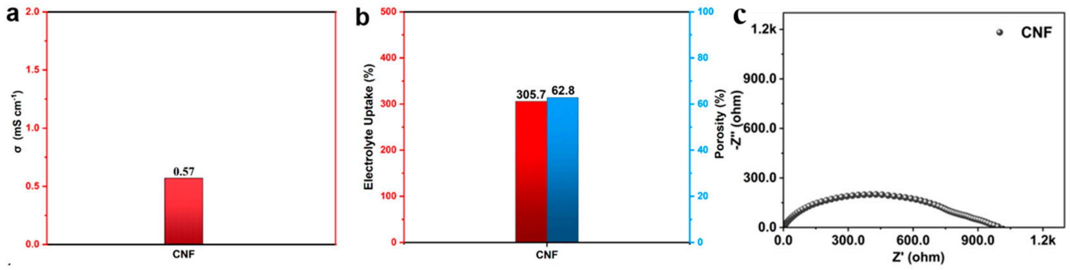

Figure S4. (a) Ion conductivity of CNF separator; (b) Porosity and electrolyte uptake of CNF separator; (c) Nyquist plot of EIS of CNF separator.

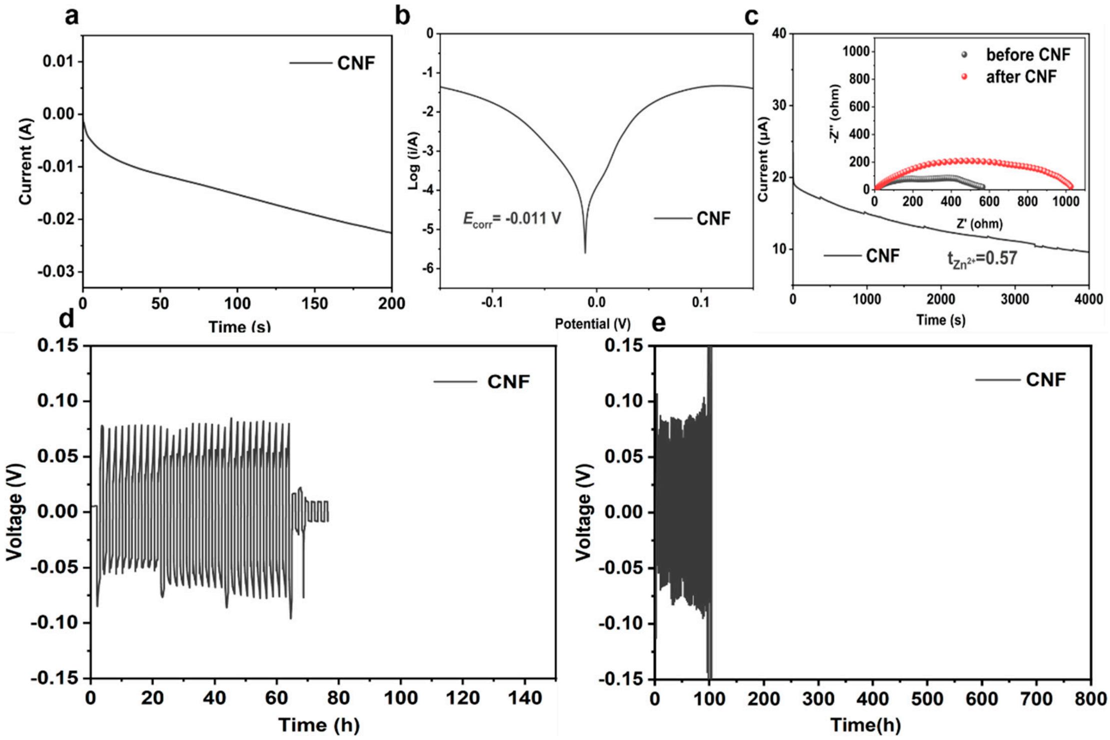

Figure S5. (a) CA curve at -150 mV overpotential of CNF separator; (b) The plot of Tafel analysis of CNF separator; (c) Zn^{2+} ion transfer number of CNF separator at 10 mV polarization voltage (the insets illustrate the changes in EIS before and after polarization); (d) Rate performance of CNF separator of Zn//Zn symmetric cells at various current densities; (e) Cycling performance of CNF separator of Zn//Zn symmetric cells at a current density of 1 mA·cm^{-2} with an areal capacity of 1 mAh·cm^{-2}.

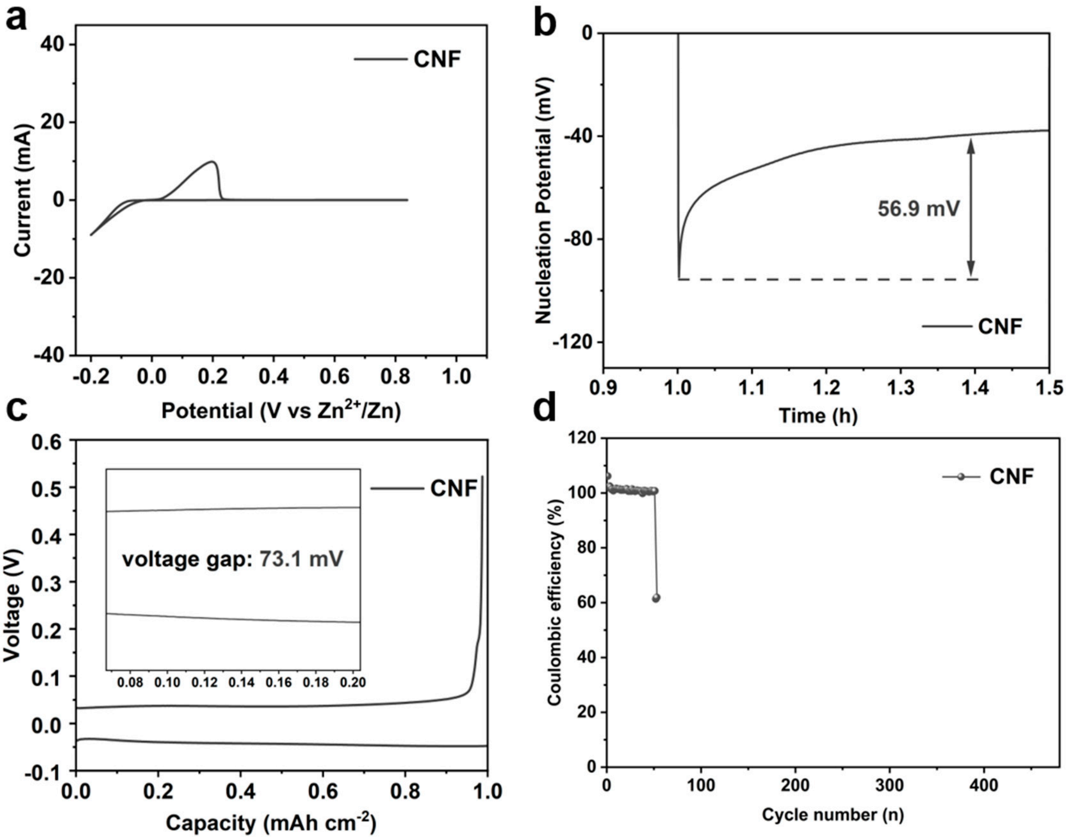

Figure S6. Electrochemical behaviors of Zn//Cu asymmetric cell with CNF separator; (a) CV curve at a scan rate of 1 mV·s^{-1}; (b) Nucleation overpotential curve at a current density of 1 mA·cm^{-2} with an areal capacity of 1 mAh·cm^{-2}; (c) Voltage-capacity profiles at a current density of 1 mA·cm^{-2} with an areal capacity of 1 mAh·cm^{-2} (the inset displays an amplified voltage gap from 0.07 to 0.2 mAh·cm^{-2}); (d) Coulombic efficiency at a current density of 1 mA·cm^{-2} with an areal capacity of 1 mAh·cm^{-2}.

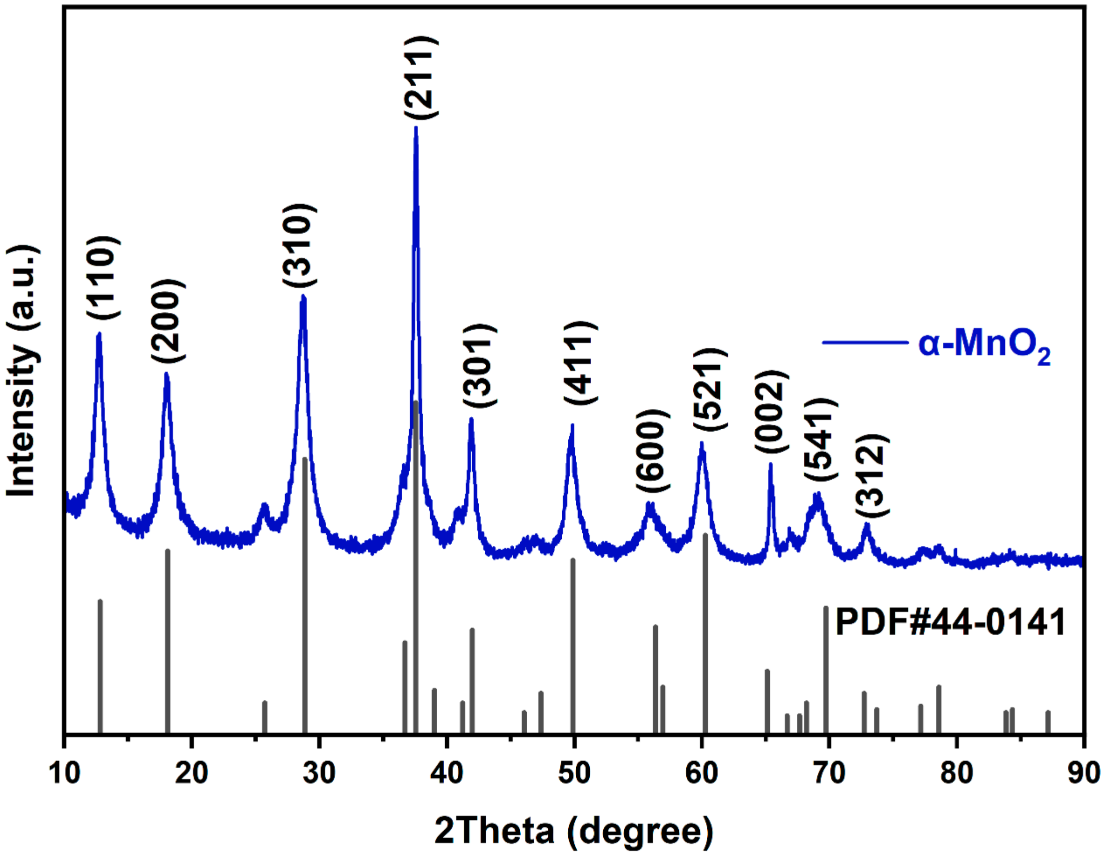

Figure S7. XRD pattern of α-MnO_2
